# COGNITIVE AND PRACTICE EFFECTS OF IMMERSIVE VIRTUAL REALITY USE IN OLDER ADULTS: PRELIMINARY RESULTS

**DOI:** 10.1093/geroni/igae098.3939

**Published:** 2024-12-31

**Authors:** Ferdinand Delgado, Jeremiah Greenberg

**Affiliations:** University of New Hampshire, University of New Hampshire, New Hampshire, United States; University of New Hampshire, Durham, New Hampshire, United States

## Abstract

Virtual reality through head-mounted displays (VR-HMD) is emerging as a tool for cognitive training in older adults. However, little is known about the impact of commercially available VR-HMD games on cognitive function. The purpose of this study was to examine preliminary results on the practice effects of VR-HMD games and their influence on cognition. Six participants (aged 71-81 years; 5 males) played VR-HMD games twice a week for eight weeks, for 60 minutes each session. Using the Meta Quest 3, participants played First Steps, featuring three laser shooting tasks (single-laser, dual-laser, and automatic laser) on weeks 1 and 5, and Fruit Ninja (Classic and Zen modes) on weeks 2 and 6. A Tai Chi and table tennis game were also played, but game scores were not recorded. Their average game score after each week was used for analysis. NeuroTrax BrainCare Tests were used to measure executive function (EF), information processing speed (IPS), attention, and visual-spatial ability before and after the intervention. Paired sample t-tests revealed significant practice effects in the single-laser (4251.24±1114.80 to 5066.36±1553.75, p=0.02) and automatic laser (4930.24±1311.34 to 6059.54±1219.53, p=0.018) tasks in First Steps, and Fruit Ninja’s classic mode (30.46±5.80 to 43.48±12.07, p=0.017). Visual-spatial ability showed a significant improvement (124.22±11.08 to 129.5±9.23, p=0.014), but not the other cognitive domains (p≥0.05). Improvement in Fruit Ninja’s classic mode showed a correlation trend with the change in visual-spatial ability (p=0.052). In conclusion, older adults can improve game performance in VR-HMD. Further research is needed to clarify VR-HMD effects on cognitive function.

